# Clinical and molecular evaluations of siblings with “pure” 11q23.3-qter trisomy or reciprocal monosomy due to a familial translocation t (10;11) (q26;q23.3)

**DOI:** 10.1186/s13039-014-0101-8

**Published:** 2014-12-24

**Authors:** Rongyu Chen, Chuan Li, Bobo Xie, Jin Wang, Xin Fan, Jingsi Luo, Xuyun Hu, Shaoke Chen, Yiping Shen

**Affiliations:** Department of Genetic and Metabolic Central Laboratory, Guangxi Maternal and Child Health Hospital, No59, Xiangzhu Road, Nanning, China; Department of Laboratory Medicine, Boston Children’s Hospital, 300 Longwood Avenue, Boston, MA 02115 USA; Department of Pathology, Harvard Medical School, 300 Longwood Avenue, Boston, MA 02115 USA

**Keywords:** Jacobsen syndrome, 11q23.3-qter trisomy, 11q23.3-qter monosomy, SNP array, Familial translocation

## Abstract

11qter trisomy is rare, mostly occurs in combination with partial monosomy of a terminal segment of another chromosome due to unbalanced segregation of parental translocations. Pure 11qter trisomy is rarer, only five cases have so far been reported. Here we report a family with all four siblings affected with neurodevelopmental disorders and facial dysmorphism. Chromosomal microarray analysis (CMA) identified 11q23.3-qter (15.1 Mb) deletion in one and reciprocal duplication in the other three siblings. Both father and grandfather are balanced translocation (46, XY, t (10;11) (q26;q23)) carriers. The genetic material involved on chromosome 10 is very limited (270 kb). Thus, the pedigree presented rare cases with “pure” 11qter trisomy or reciprocal 11qter monosomy (Jacobsen syndrome), offering a unique opportunity to examine clinical presentations of multiple individuals with identical genomic imbalance.

The proband with 11qter monosomy presented with many features of Jacobsen syndrome. The three 11qter trisomy carriers presented with shared craniofacial features including brachycephaly and short philtrum. They are also significant for the following neurodevelopmental and neuropsychiatric defects: intellectual disability, expressive language deficiency, autistic features, auditory hallucination, self-talking and pain insensitivity. To our knowledge, this is the smallest “pure” trisomy 11qter so far reported and this is the first report to describe the neuropsychiatric features of patients with 11qter trisomy. Our observation also revealed dissimilar features in our patients compared with those of previously published trisomy 11qter cases. The pedigree also revealed phenotypic heterogeneity among siblings with identical genomic imbalance.

## Background

Monosomy 11qter causes Jacobsen syndrome (Jacobsen, 1973; OMIM 147791), a rare but clinically well-known genomic disorder that has an incidence of approximately 1 in 100,000 births [1]. Several hundred cases of Jacobsen syndrome have been reported in the literature. Trisomy 11qter, initially described in 1977 [2], is rarer and less well characterized. Pihko et al., reviewed 20 cases published prior to the year 1981 while presenting a new case of their own,19 out of 21 trisomy 11qter cases occurred due to unbalanced segregation of parental translocations [3]. In 13 cases with 46 chromosome composition, monosomy on another chromosome was also present. Seven out of the remaining eight cases with 47 chromosome composition were Emanuel syndrome. Emanuel syndrome, also called derivative chromosome 22, is caused by an extra chromosome due to a 3:1 meiotic malsegregation of a parental translocation t (11;22) (q23.3;q11.23), which leads to offspring with trisomy 11q23.3-qter and an extra copy of the top and middle part of chromosome 22. The phenotype of Emanuel syndrome is believed to be caused by the extra copies of genes on both chromosome 22 and 11qter. None of the cases reported by Pihko et al. was pure trisomy 11qter. Since then, more than thirty 11q distal trisomy (involving telomere) cases other than the Emanuel syndrome have been reported in the literature [4-22] and the Decipher database (case# 282318; 251238; 258577; 251238 and 253250) (https://decipher.sanger.ac.uk). Common clinical features of these cases are similar to that of Emanuel syndrome, which include pre- and post-natal growth retardation, psychomotor delay, mild to moderate intellectual disability, distinctive craniofacial abnormalities such as microcephaly, brachycephaly, a short nose; low-set ears, high arched/cleft palate, preauricular pits or tags, micrognathia and long philtrum. In addition, congenital heart defects, hypoplasia or agenesis of the corpus callosum and in affected males, cryptorchidism and micropenis, have also been observed. Thus it is believed that 11qter trisomy contributed to most of the common features. However because of the involvement of subtelomeric imbalances of other chromosomes, it is still difficult to assign particular features to 11qter trisomy.

Few cases showed “pure” 11qter trisomy without significant involvement of another chromosome. These cases provided opportunity for analyzing the clinical consequence of trisomy 11qter. Currently, only five such cases have been previous reported [5,8,12,17,22]. Here we presented the molecular and clinical features of three siblings with the smallest “pure” 11qter trisomy.

## Case presentation

**Figure 1:**
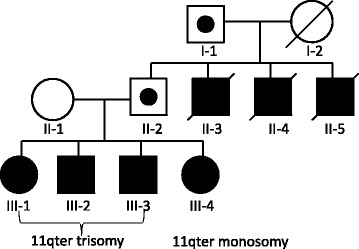
**The pedigree.** The proband (III-3) has two sisters (III-1, 4) and one brother (III-2). The proband’s father (II-2) and grandfather (I-1) were the balanced translocations carriers without clinical presentations. Three uncles (II3-5) also had intellectual disability and all of them died at the age of 17–20 years old.

**Figure 2:**
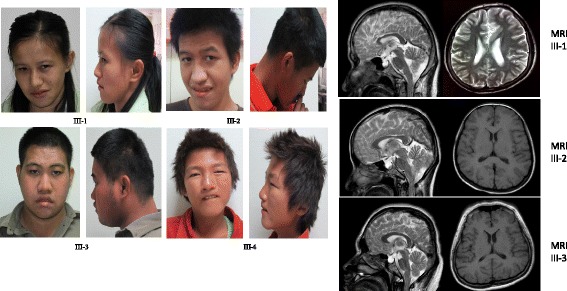
**Clinical features of the patients.** Left panel: III1-3: The craniofacial features of trisomy 11q23.3-q25 patients. Multiple facial dysmorphisms including microcephaly, brachycephaly, plagiocephaly and short philtrum are shown. III-4: The craniofacial features of the patient with Jacobsen syndrome. Her craniofacial features include facial asymmetry, low set ears, blepharophimosis, ptosis and epicanthal folds, broad nasal bridge, a thin upper lip. Right Panel: The brain MRI of 11q23.3-q25 trisomy patients. The brain MRI indicates the small pituitary gland and empty sella, and the bilateral parietal sulcus deepening was observed.

We identified a family with 4 affected siblings (Figure 1). The proband (III-3) has two sisters (III-1, 4) and one brother (III-2), all of them are clinically affected with intellectual disability, craniofacial dysmorphism and neuropsychiatric problems (Figure 2). The proband’s father (II-2) and grandfather (I-1) were the balanced translocations carriers without clinical presentations. Three uncles (II3-5) also had intellectual disability and all of them died at the age of 17–20 years.

**Patient 1** is the proband (III-3), a 19-year-old boy. He was born after an uneventful pregnancy (G3P3) and delivered at full term. He had somewhat normal early development but regression was noticed around age of 4. He was reported to have significant mental decline around age 7. He recently had a seizure and was hospitalized for losing consciousness. Laboratory examinations of complete blood count, TSH and IGF-1 were normal, cardiac ultrasound and hearing tests were unremarkable. He was diagnosed with a lung infection and hydrothorax. In addition, electroencephalogram examination showed abnormal signal. Brain MRI examination revealed the following abnormalities: bilateral hippocampal atrophy, ventriculomegaly, thinning of corpus callosum, hemiseptum cerebri and empty sella. Brain Magnetic Resonance Angiogram (MRA) was normal. The boy was evaluated at the genetics clinic for the first time when he was at the age of 19 years. Physical examination showed normal muscle tone, his height, weight and occipitofrontal circumference were 168 cm (−0.77SD), 59 kg (0.23SD) (BMI = 20.9 kg^2^/cm) and 54 cm (23%) respectively. His craniofacial features include, brachycephaly, long face, bitemporal narrowing, upslanted palpebral fissures, deep-set eye, big nose, thick up-lip and short philtrum. He has severe intellectual disability and speech deficit. He is insensitive to pain. He has no eye contact or social communication and does not response to instructions. He is constantly moving his legs and hands. His repetitive and stereotypic activities include patting the chair, clapping hands and rubbing his hands against his leg. He met the clinical diagnostic criteria of autism spectrum disorder.

**Patient 2** is a 23-year-old girl (III-1) evaluated at the genetics clinic for the first time. She was born after an uneventful pregnancy and delivered at full term. Her cardiac ultrasound and hearing test results were normal. Brain MRI showed a small pituitary gland and empty sella. Her height, weight and occipitofrontal circumference at the age of 23 were 148 cm (−2.31SD), 36 kg (−3.00SD) (BMI = 16.4 kg^2^/cm) and 48 cm (−6 s.d.) respectively. She had some similar craniofacial features as the proband including severe microcephaly, brachycephaly, long face, upslanted palpebral fissures, deep-set eye, full up-eyelid, big nose and short philtrum. She has learning disability with poor academic performance at school. She has normal language and social communication skills. She takes care of herself and her brothers. She has had normal menstruation. She is insensitive to pain. She has auditory hallucination and often exhibits self-talking behavior. She was diagnosed with schizophrenia and has been treated with schizophrenia medication.

**Patient 3** is a 23-year-old boy (III-2), he was born after an uneventful pregnancy and delivered at full term. His birth weight was in the normal range with no feeding difficulty. He was noticed to have significant cognitive decline at 7 years old as indicated by his school performance. He was evaluated in the genetics clinic for the first time at 21 years of age, his height, weight and occipitofrontal circumference were 159 cm (−2.30SD), 85 kg (+2.00SD) (BMI = 30.1 kg^2^/cm) and 55 cm (47%) respectively. He had very similar craniofacial features and neurobehavioral issues as the proband including autistic features. In addition, he presented with branchydactyly, obesity and acanthosis nigricans. He has micropenis and cryptorchidism. He is also insensitive to pain. He had a normal cardiac ultrasound and hearing exam.

**Patient 4** is a 16-year-old girl (III-4). She was born at full term following an obstructed labor. She walked at the age of 4–5 years. She had no feeding difficulty. Her intellectual disability was noticed at early childhood. At the age of 16, she was evaluated for the first at the genetics clinic. Her height, weight and occipitofrontal circumference were 141 cm (−3.51SD), 32 kg (−3.75SD) (BMI = 16.1 kg^2^/cm) and 52 cm (2%, −2.2 s.d.) respectively. She presented with severe speech deficit (only vocalize two-words sentences with simple pronunciation), but is reactive with surroundings and responses to instructions. Her craniofacial features include borderline microcephaly, facial asymmetry, low set ears, down-slanted palpebral fissures, blepharophimosis, ptosis and epicanthal folds, broad nasal bridge, downturned corners of the mouth and a thin upper lip. She had a normal philtrum. Unlike her other siblings, her hair color is much lighter. She also presented with pachyglossia, interphalangeal joint finger stiffness, and gait abnormalities. Frequent Nose bleeding and pain insensitivity were observed, laboratory examinations of complete blood count, TSH and IGF-1 were normal, cardiac ultrasound and hearing tests were normal.

### Chromosomal microarray (CMA) analyses

**Figure 3:**
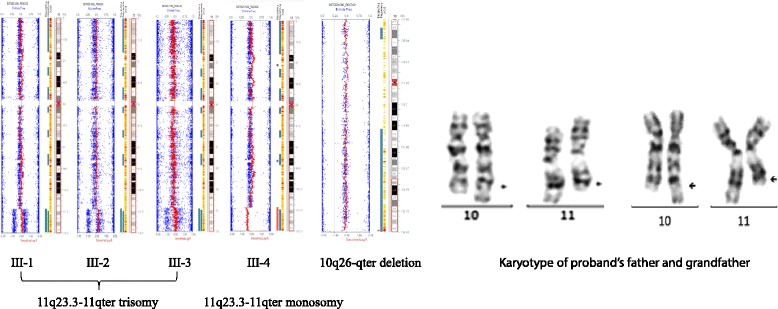
**The results of chromosomal microarray analysis and karyotyping.** Left panel: CMA scatter plot showing identical duplications in patient III-1-3 and reciprocal deletion in patient III-4 at 11q23.3-11qter. A small telomeric deletion of 10q26-qter detected in patient III-1-3. Right panel: karyotype showing the two chromosomes involved with translocation in proband’s father and grandfather.

DNA samples were extracted from peripheral blood using Lab-Aid DNA kit (Zeesan Biotech Co., Ltd, China), genomic profiling was performed using the illumina Human SNP cyto-12 array, which includes over 300 k single nucleotide polymorphisms (SNPs) in the human genome. Hybridization and array scanning were performed according to the manufacturer’s instruction. Data were analyzed with Illumina Genome Studio and KaryoStudio software. The segment report was restricted to regions of 100 kb or greater with 10 or more consecutive probes that differed significantly from the expected normalized diploid values. CMA analyses revealed a 15.1 Mb terminal duplication at the region of 11q23.3-11qter (chr11:119842708–134944006, NCBI build 37) in patients 1–3 and a reciprocal deletion in patient 4. A 270 Kb terminal deletion at 10q26 chr10:135153849–135430043, NCBI build 37) was also detected in patients 1–3 and a reciprocal duplication in patient 4 (Figure 3A).

### Cytogenetic analyses

Karyotyping analysis was performed using G-banding techniques on stimulated blood lymphocytes and analyzed at 400–500 band resolution. G banding of the parents, at a resolution of at least 400 bands per haploid genome, the cytogenetic analysis of the father revealed a balanced translocation between the chromosome 10 and chromosome 11, the Karyotype of the proband’s father and grandfather were 46, XY, t (10;11) (q26;q23), the mother showed a normal Karyotype (Figure 3B). Thus, patient 1–3 have 11qter trisomy and patient 4 has 11qter monosomy (Jacobsen syndrome).

## Conclusion

**Figure 4:**
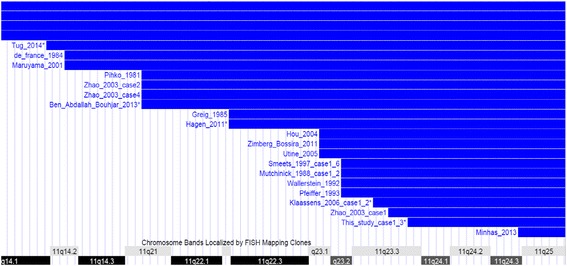
**Custom tracks showing the locations and relative sizes of previously published 11q duplications.** *Denote the duplication in our patient III-1-3.

11qter trisomy often co-occur with genomic imbalances of another chromosome, most often trisomy 22 in cases of Emanuel syndrome and monosomy of other chromosome in other cases due to parental translocations. Patients with “pure” 11qter trisomy are rare but provide opportunity for evaluating the clinical consequences of increased dosage of genes on the distal portion of 11q. So far, only five cases with “pure” 11qter trisomy have been described. Greig et al., in 1985 reported a male infant with 11q22-qter duplication due to a parental translocation between 11q and 9p. Although the 9 p was involved in the balanced parent, no apparent deletion was observed, thus this was considered as a “pure” 11qter trisomy case by the author [5]. Pfeiffer and Schutz reported a dysmorphic child with a tandem duplication of 11q23-qter which was inherited from a mosaic mother with the same duplication [8]. Zhao et al., reported a 5-years-old girl with an inverted duplication of 11q13-qter as the only cytogenetic abnormality detected in the patient [12]. Recently, a 17 month old boy with “pure” 11q23.1-qter trisomy [17] and a 10-month-old boy with 11q14.1-qter trisomy and minimal involvement of 12p [22] were reported. Among the above five cases, only the most recent case by Tug et al. was molecularly evaluated by chromosomal microarray, where the exact range of 11q duplication and the size and gene content of 12p monosomy were determined. Here we reported three siblings with distal 11q duplication and a small deletion on 10qter due to a familial balanced translocation between 10q and 11q. We used high resolution microarray, delineated the breakpoint of the duplication and deletion. The 276Kb deletion on chromosome 10 contains about 10 Refseq genes (*PRAP1, FUOM, ECSH1, PAOX, MTG1, SPRN, SCART1, CYP2E1, SYCE1* and *SPRNP1*). None of them are known to be associated with human diseases. The region is highly copy number polymorphic as reported in DGV (http://dgv.tcag.ca/dgv/app/homeAccessedSept.2014.). Thus we believe the involvement of 10q deletion is minimal in this case and our patients presented another case of “pure” 11qter trisomy. We determined that the 11qter trisomy detected in our patients is the smallest among those with “pure” 11qter trisomy, it is also one of the smallest among all cases with 11qter trisomy (Figure 4). We compared the clinical phenotypes of “pure” 11qter trisomy cases (Table 1).

**Table 1:** **Clinical features of patients with “pure” 11qter trisomy**

	**Greig, 1985** **[** 5 **]**	**Pfeiffer, 1993** **[** 8 **]**	**Zimberg, 2011** **[** 17 **]**	**Tug, 2014** **[** 22 **]**	**Zhao, 2003** **[** 12 **]**	**Patient 1**	**Patient 2**	**Patient 3**
**Head/neck**	Microcephaly, large anterior fontanelle	Microcephaly, *brachycephaly*	Microcephaly, *brachycephaly*, short neck	*Brachycephaly,* relative microcephaly, short neck	Microcephaly, *plagiocephaly*	*brachycephaly*	Microcephaly, *brachycephaly*	*brachycephaly*
**Face**	Sparse eyebrows,micrognathia, low set ears, large ears, low hairline	Hypertelorism, large eyes, **small nose**, high palate, large ears,microstomia,Micrognathia, long eye lashes, strabismus	Broad nasal base, anteverted nares, **long philtrum**, low set ears	Sparse eyebrows, high palate, short and upslanting palpebral fissures, **thin lips**, broad nasal base, micrognathia, asymmetric and posteriorly rotated ears, uplifted ear lobules	Round face, Broad nasal base, small nose, bulbous nasal tip, high palate,microstomia ,micrognathia, ,myopia, colobomas, preauricular sinus	Long face, bitemporal narrowing, upslanted palpebral fissures, deep-set eye, full up-eyelid, thick and up-lifting eyebrow, **big nose**, **thick up-lip** and **short philtrum**	Long face, upslanted palpebral fissures, deep-set eye, full up-eyelid, **big nose** and **short philtrum**	Long face, bitemporal narrowing, upslanted palpebral fissures, deep-set eye, full up-eyelid, thick and up-lifting eyebrow, **big nose**, **thick up-lip** and **short philtrum**
**Cardio-vascular**	**—**	ASD	ASD, pulmonary stenosis	ASD/PDA, right aorticarch, aberrant left subclavianartery	ASD, malformed epiglottis, tracheomalacia	**—**	**—**	**—**
**Skeletal**	Clinodactyly and syndactyly of fingers	NR	Scoliosis, tethered spinal cord, hip islocation	Short and broad halluces	Short limbs, brachydactyly, asymmetric leg lengths	**—**	**—**	**—**
**Skin, nails, hair**	Single palmar crease	NR	NR	Single palmar crease,Widely spaced-invertednipples, Sacral dimple, hypoplastic toenails	Single palmar crease	Nevi on the neck and face	Nevi on the neck and face	Nevi on the neck and face, acanthosis nigricans,
**Abdomen**	**—**	Inguinal hernia	Inguinal hernia,Diaphragmatic hernia	**—**	GER, feeding intolerance	**—**	**—**	**—**
**Genito-urinary**	Micropenis, Hypospadias	**—**	Micropenis/cryptorchidism,Unilateralrenalagenesis	Cryptorchidism	**—**	**—**	**—**	Micropenis/cryptorchidism
**Neurological**	Hypotonia,Intellectual seizure/disability,blindness	Intellectual disability,Brain malformation	Hypertonicity,Intellectual disability/seizure, Sensorineural hearing loss	Intellectual disability,Sensorineural hearing loss,Dilatation of third-lateral ventricles, thin corpus callosum	Hypotonia, Intellectual disability, seizure	Intellectual disability,hypalgesia, abnormal brain imaging, seizure, autism spectrum disorder	Intellectual disability,hypalgesia,abnormal brain imaging, schizophrenia	Intellectual disability,seizures, hypalgesia,abnormal brain imaging, autism spectrum disorder

**—**: Feature absent NR: Not recorded. ASD: atrial septal defect; VSD: ventricular septal defect. Italicized features are consistent in our patients, as well as with previously reported patients. Bolded features are consistent among our patients but opposite to some previously described patients.

Based on the comparison, we noticed that patients with “pure” 11qter trisomy exhibited some similar craniofacial features including brachycephaly or plagiocephaly which are also commonly observed in Emanuel syndrome patients. Other features that are frequently reported in previous reported patients with “pure” 11qter trisomy or Emanuel syndrome such as micrognathia, high arched/cleft palate, preauricular pits or tags are not present in our patients. Short philtrum, big nose and thick lip are consistent features among the siblings with trisomy 11qter in this family, these features are the opposite to what were described for some of the previous 11qter trisomy patients. Three siblings in this family showed short stature and it is not known if growth retardation is a common feature for “pure” trisomy 11qter patients since the height information was very limited for previously described patients. Male reproductive system abnormalities such as micropenis or cryptorchidism are common but do not occur in every male patient. Two brothers in this family were discordant for these features. Congenital heart defects had been reported in most of previous patients, whereas our patients do not appear to have any structural heart defects. Neurologically, all patients showed intellectual disability although the severity differs. They are all insensitive to pain. It is interesting to note that the two male siblings in this family exhibited severe intellectual disability, whereas the sister who carried the same 11qter duplication only exhibited learning disability. Our patients have some consistent brain imaging abnormality and it is yet to be seen if these are common features for patients with 11qter trisomy. Seizures are common but not present in all. Based on these observations, it seems that brachycephaly and intellectual disability are consistently associated with 11qter duplication. Brain abnormalities, seizure and reproductive system anomalies are common but not fully penetrant.

The most important novel clinical features that we observed in our patients are severe neuropsychiatric issues. In particularly, all three 11qter trisomy patients have very limited social communication and social reciprocity. They almost always look down to the floor and do not look directly to people. All of them have self-talking behaviors. Two boys have significant stereotyped and repetitive motor movements. Both lack of any spoken language and do not follow instructions. They met the clinical diagnostic criteria for autism spectrum disorder. Interestingly, the female trisomy patient has good language skill and good response to instructions. Her behavior issues are much less severe. This family provided an interesting scenario that same genomic imbalance lead to different ASD phenotype in male and female. The female is less affected than male, which is consistent with the “female resistant” theory of autism [23].

Recently, the behavioral characteristics of 17 patients with 11qter deletion (Jacobsen syndrome) were evaluated and 8 (47%) of them were found to have behavioral features consistent with autism spectrum disorders [24]. We reported here for the first time that “pure” 11qter trisomy may also increase the risk of autistic features. The likely reason that the behavioral features were not well described in previous patients is due to the fact that the majority of previous patients were at a very young age at the time of examination. Full features of ASD were not present. Recently, Minhas et al., reported a 10q monosomy and 11qter trisomy in a pair of boys at 7 and 5 years old with ASD, the authors attributed the ASD features to the 11ter duplication [25]. More patient evaluation is required to assess the association of 11qter trisomy and autism spectrum disorder.

In summary, we reported the clinical characteristics of siblings carrying a rare “pure” 11qter trisomy, one of the smallest so far reported. In addition to the craniofacial features, these patients are found to share some neurodevelopmental and neuropsychiatric defects including intellectual disability, autistic features, auditory hallucination and pain insensitivity.

## Consent

Written informed consent was obtained from the parents of the proband for publication of this Case Report and any accompanying images. The consent form was approved by the ethical committee of Guangxi Maternal and Child Health Hospital, China. A copy of the written consent is available for review by the editor of this journal.
